# Transcriptome Analysis Reveals Differentially Expressed Genes and Long Non-coding RNAs Associated With Fecundity in Sheep Hypothalamus With Different FecB Genotypes

**DOI:** 10.3389/fcell.2021.633747

**Published:** 2021-05-20

**Authors:** Si Chen, Xiaofei Guo, Xiaoyun He, Ran Di, Xiaosheng Zhang, Jinlong Zhang, Xiangyu Wang, Mingxing Chu

**Affiliations:** ^1^Key Laboratory of Animal Genetics, Breeding and Reproduction, Ministry of Agriculture and Rural Affairs, Institute of Animal Science, Chinese Academy of Agricultural Sciences, Beijing, China; ^2^Tianjin Institute of Animal Sciences, Tianjin, China

**Keywords:** hypothalamus, FecB, lncRNA, sheep, fecundity, follicular development

## Abstract

Small-tailed Han sheep, with different *FecB* genotypes, manifest distinct ovulation rates and fecundities, which are due to differences in reproductive hormones secreted by the hypothalamic–pituitary–ovarian axis. Nevertheless, the function of the hypothalamus against a *FecB* mutant background on increasing ovulation rate is rarely reported. Therefore, we determined the expression profiles of hypothalamus tissue collected from six wild-type (WW) and six *FecB* mutant homozygous (BB) ewes at the follicular and luteal phases by whole-transcriptome sequencing. We identified 53 differentially expressed mRNAs (DEGs) and 40 differentially expressed long non-coding RNAs (DELs) between the two estrus states. Functional annotation analysis revealed that one of the DEGs, PRL, was particularly enriched in the hypothalamic function, hormone-related, and reproductive pathways. The lncRNA–target gene interaction networks and KEGG analysis in combination suggest that the lncRNAs LINC-676 and WNT3-AS *cis-*acting on *DRD2* and *WNT9B* in different phases may induce gonadotropin-releasing hormone (GnRH) secretion. Furthermore, there were differences of regulatory elements and WNT gene family members involved in the follicular–luteal transition in the reproductive process between wild-type (*WNT7A*) and *FecB* mutant sheep (*WNT9B*). We combined the DEG and DEL data sets screened from different estrus states and genotypes. The overlap of these two sets was identified to select the mRNAs and lncRNAs that have major effects on ovulation. Among the overlapping molecules, seven DEGs and four DELs were involved in the follicular–luteal transition regulated by *FecB* mutation. Functional annotation analysis showed that two DEGs (*FKBP5* and *KITLG*) were enriched in melanogenesis, oxytocin, and GnRH secretion. LINC-219386 and IGF2-AS were highly expressed in the BB ewes compared with WW ewes, modulating their target genes (*DMXL2* and *IGF2*) to produce more GnRH during follicular development, which explains why mutated ewes produced more mature follicles. These results from expression profiling of the hypothalamus with the *FecB* mutation at different estrus states provide new insights into how the hypothalamus regulates ovulation under the effect of the *FecB* mutation.

## Introduction

The *FecB* gene was first identified to have a major effect on the ovulation rate and small size of the Booroola Merino (Davis et al., 1982). Subsequently, three studies revealed that the *FecB* mutation (A746G) in the coding region of the *BMPR1B* gene on sheep chromosome 6 resulted in the substitution of one amino acid (arginine to glutamine, Q249R) in its protein sequence (Mulsant et al., 2001; Souza et al., 2001; Wilson et al., 2001). The mutation at this site can weaken the function of ligands involved in BMP signal transduction, inhibiting the proliferation but promoting the differentiation of granulosa cells, which leads to the accelerated maturation and ovulation of some oocytes (Fabre et al., 2006; Kumar et al., 2020). The effect of the *FecB* gene is additive for ovulation rate and partially dominant for litter size (Qi et al., 2020), including in small-tailed Han sheep (Chu et al., 2007). The ovulation rate and litter size of *FecB* mutant homozygotes were found to be significantly greater than those of *FecB* wild type (Chu et al., 2011).

Strict regulation of the central nervous system and endocrine system is essential for reproductive hormone synthesis and release by the hypothalamic–pituitary–ovarian axis (Abreu and Kaiser, 2016). The hypothalamus secretes gonadotropin-releasing hormone (GnRH), which triggers the secretion of luteinizing hormone (LH) and follicle-stimulating hormone (FSH) from the pituitary gland. FSH and LH serve as regulators of the ovary by driving folliculogenesis, estrogen (E2), and progesterone (P4) in the estrus cycle (McNeilly and Fraser, 1987). That is, GnRH, an initiator of reproduction, performs basic roles in the modulation of reproductive process. A previous study revealed the relationship between the mutation and putative transcriptional changes in the hypothalamus, indicating that genes involved in the endocrine system, such as prostaglandin-endoperoxide synthase 2 (*PTGS2*) in the ovarian steroidogenesis pathway, are expressed to a greater extent in the hypothalamus of the sheep heterozygous for the *FecB* mutation than in wild-type ewes (Tian et al., 2020).

Gonadotropin-releasing hormone, FSH, and LH are proteins whose expression is predominantly dictated at the transcriptional and post-transcriptional levels during the formation and release of these hormones (Li et al., 2019). That is, diverse types of non-coding RNA, including microRNAs (miRNAs), lncRNAs, and circular RNAs (circRNAs), have important effects on modulating hormones via the hypothalamic–pituitary–ovarian axis. Some studies reveal that lncRNAs participated in the reproductive process (Miao et al., 2016, 2017; Feng et al., 2018). Neuronal tyrosine phosphorylated phosphoinositide 3-kinase adaptor 1 (NYAP1) and BCL6 corepressor like 1 (BCORL1) are shown to be involved in ovary development by modulating the target genes related to the oxytocin signaling pathway (Miao et al., 2016). In addition, XLOC_446331 is suggested to potentially regulate female puberty (Gao et al., 2018). LNC_000073 and LNC_000888 target the genes phosphatidylinositol-4,5-bisphosphate 3-kinase catalytic subunit alpha (PIK3CA) and fos proto-oncogene, ap-1 transcription factor subunit (FOS) that modulate estrus, respectively (Li et al., 2019). Furthermore, hypothalamic lncRNA GnRH-E1 RNA might be involved in GnRH neuron development and maturation by inducing the transcriptional activity of *GnRH1* gene regulatory element (Huang et al., 2016).

The studies performed to date have mainly focused on the functional role of lncRNAs in ovarian tissue, so little is known about their role in the hypothalamus and their mechanism of regulating reproductive hormone activity in sheep. Identification of key regulators against a background of *FecB* mutation that affects follicular development in the hypothalamus could be beneficial for understanding the mechanisms, by which the *FecB* gene regulates follicular development and ovulation at the molecular level and for the screening of candidate protein-coding genes associated with the reproductive ability of ewes. In the present study, we performed differential expression analysis and gene function analysis using RNA-seq and established lncRNA–mRNA interaction networks to explore the role of hypothalamic lncRNAs and genes in a context in which *FecB* mutation affects follicular development.

## Materials and Methods

### Ethics Statement

All the ewes involved in this experiment were supported by the Science Research Department of the Institute of Animal Sciences, Chinese Academy of Agriculture Sciences (IAS-CAAS). In addition, Ethical Approval was in compliance with the Animal Ethics Committee of the IAS-CAAS (No. IAS 2019-49).

### Animals and Tissue Samples

Based on TaqMan assays (Liu et al., 2017), six wild-type (WW) and six *FecB* mutant homozygous (BB) small-tailed Han sheep were selected from a nucleus herd in the southwest of Shandong Province, China. All non-pregnant ewes, approximately 3–4 years old with similar weights, were provided with free access to food and water under natural temperature and lighting conditions.

Before the experiment, all selected ewes were treated with vaginal sponges (InterAg Co., Ltd., New Zealand) (progesterone 300 mg) and injected with vitamin AD to protect the endometrium. The vaginal sponges were removed after 12 days with the time of removal being set as 0 h. These ewes were divided into follicular and luteal phase groups. Then, six ewes (three BB ewes and three WW ewes) were euthanized at 45 h (follicular phase). Six ewes (three BB ewes and three WW ewes) were euthanized at 216 h (luteal phase). Hypothalamus samples were dissected immediately after euthanasia, and the 12 tissue samples were frozen in liquid nitrogen and stored at −80°C until further analysis.

### RNA Isolation, Library Construction, and Sequencing

Total RNA from the hypothalamus was extracted using TRIzol reagent (Thermo Fisher Scientific, Waltham, MA, United States). RNA purity was checked with a Nano Photometer^®^ spectrophotometer (IMPLEN, Westlake Village, CA, United States), and its concentration was measured with Qubit^®^ RNA Assay kits (Thermo Fisher Scientific). RNA integrity was assessed with RNA Nano 6000 Assay, with the RNA integrity number (RIN) value of all samples being greater than seven.

Three micrograms of total RNA were taken as a starting amount to construct a lncRNA library. To remove ribosomal RNA (rRNA) from the sample, Ribo-Zero^TM^ GoldKits (Epicentre, United States) were used to digest the total RNA. In addition, in accordance with the manufacturer’s instructions for the NEB Next Ultra Directional RNA Library Prep Kit for Illumina (NEB, Ipswich, United States), RNA-sequencing libraries were generated by paired-end sequencing. Subsequently, the pooled libraries were sequenced on Hiseq X (Illumina, San Diego, CA, United States), using a chain-specific library construction strategy to count the number and types of various transcripts (mRNAs, known lncRNAs and novel lncRNAs). All sequencing data was outsourced to Annoroad Gene Technology Co., Ltd. (Beijing, China).

### Data Quality Control and Assembly

We used bcl2fastq (v2.17.1.14) to deal with Illumina high-throughput sequencing, converting original image files into raw sequenced reads on base calling, which were raw reads. The raw reads were filtered using in-house Perl scripts made by Annoroad Gene Technology Co., Ltd. (Beijing, China), including removing reads with adapter contamination, low-quality reads (Phred Quality Score <5%), reads with poly-N >5%, and reads that matched rRNA (Sulayman et al., 2019). The Phred quality score represented the rate of different base sequencing error, such as Q20 and Q30 indicating that the base sequencing error rate is 1% and 0.1%, respectively. Then, the clean data, obtained through repeated testing, were mapped and assembled using HiSAT2 (v2.0.5) (Pertea et al., 2016) and String Tie (v1.3.2d) (Pertea et al., 2015), respectively, based on the sheep reference genome (Ovis_aries.v4.0_2). HiSAT2 was run with “–rna-strandness RF” and “–dta -t -p 4,” String Tie with “-G ref.gtf -rf -1,” and the other parameters were set as the default. According to the sheep gene annotation from relevant databases, the number and proportion of three gene functional elements (exon, intron, and intergenic) were determined on the unique mapping sequences. Finally, homogeneity analysis was performed on the sequences to ensure that the sequencing results did not affect the further transcriptome analysis results.

### Identification of Potential lncRNA Candidates

To decrease the false-positive rate, the following steps were taken to identify putative lncRNAs, including lincRNAs, intronic lncRNAs, and antisense lncRNAs (Ran et al., 2016). (1) Transcripts shorter than 200 bp and a single exon were discarded. (2) Transcripts with read coverage of less than five were removed. (3) The assembled transcripts were annotated using the gffcompare program^1^ to discard known mRNAs and other ncRNAs (rRNAs, tRNAs, snoRNAs, snRNAs, etc.). (4) The remaining transcripts were identified as three kinds of lncRNAs using information on class_code^2^. (5) We determined the lncRNAs overlapping among the four coding potential analyses (with CNCI, CPC, Pfam, and CPAT) and established them as the final novel lncRNA analysis data set. The coding-non-coding index (CNCI) was used to distinguish coding–non-coding transcripts, mainly analyzing the spliced transcripts, according to the characteristics of adjacent nucleotide triplets (Sun et al., 2013). CNCI was run with “–score 0 –length 199 –exon_num 2” with the other parameters set as the default. The coding potential calculator (CPC) was used to calculate protein-coding potential, including comparing transcripts with known protein databases by blastx and evaluating transcript-coding potential based on the biological sequence characteristics of each transcript coding frame (Kong et al., 2007). In both CNCI and CPC, a score <0 was considered to indicate that the lncRNA could actually be defined as a non-coding RNA. The protein families database (Pfam) was used to search for protein domains in the Pfam HMM library to screen out transcripts with known protein domains (Finn, 2005). When the *E*-value was ≥0.001, the lncRNA could be defined as non-coding RNA. Pfam was run with “minimum protein length: 60” and the other parameters set as the default. The coding potential assessment tool CPAT (CPAT, v1.2.1) was used to screen the coding RNAs by constructing a logistic regression model and calculating Fickett score and Hexamer score, which were based on open reading frame length and coverage, respectively (Wang et al., 2013).

### Differential Expression Analysis of lncRNAs and mRNAs and Clustering

The HTSeq Python package (v0.6.1) was adopted to calculate read counts, based on the HiSAT BAM files and the reference GFT file. HTSeq was run with “-I gene_id -f bam -s” and “reverse -a 10 -q” with the other parameters set as the default. For screening out DELs and DEGs between the two comparison groups, we adopted DESeq2 package (v1.28.1; Supplementary Table 9) to calculate log_2_(fold change) and *p*-value based on the normalized counts (Love et al., 2014). For biological replicates, the criteria for screening DELs and DEGs were an absolute value of log_2_(fold change) > 1 and *p*_*adj*_ < 0.05, based on the negative binomial distribution model. According to the expression level of a single gene and the information entropy method, Jensen–Shannon divergence (JS) was calculated. The maximal specificity JS score of the gene was regarded as its tissue-specific score. String Tie (v1.3.2d) was applied to calculate the fragments per kilobase of transcript per million mapped reads (FPKM) of transcripts, which was regarded as an indicator of lncRNA and mRNA expression in each sample. Based on the log_2_(FPKM) value of each gene and lncRNA, systematic clustering analysis was performed using pheatmap (v1.0.2) (Kolde and Kolde, 2015) to explore the similarities and analyze the relationships between the different libraries. The analysis consisted of Pearson’s correlation and Euclidean distance methods.

### Potential Target Gene Prediction for Differentially Expressed lncRNAs

To explore lncRNA functions in sheep hypothalamus tissue, the relationship between lncRNAs and protein-coding genes was predicted using the distances and expressions correlation of them, namely *cis-* and *trans-*acting. For each lncRNA locus, the protein-coding genes 50 Kb upstream and downstream of it were screened out as potential *cis-*elements of lncRNAs (Zhang et al., 2019). Then, we calculated Spearman’s correlation coefficients between each lncRNA–mRNA pair based on the FPKM value. Genes having significant correlations with lncRNAs at corr > 0.95 were identified as the potential *trans-*acting target genes of the DELs (Liao et al., 2011).

### Construction of lncRNA–Target Gene Co-expression Network

To infer the functions of lncRNAs in small-tailed Han sheep hypothalamus and fecundity, we constructed networks based on complementary pairs between mRNAs and lncRNAs, both of which were the nodes in the networks. The most highly related lncRNAs and target genes were screened and visualized with Cytoscape (v3.8.0) by correlation coefficient. Cytoscape was run with “layout = ‘attribute circle layout”’ with the other parameters set as the default.

### Functional Annotation of Candidate Genes

To clarify the potential roles of the lncRNA’s target genes, Gene Ontology (GO) categories (Ashburner et al., 2000; Consortium, 2019) and Kyoto Encyclopedia of Genes and Genomes (KEGG) (Kanehisa and Goto, 2000) pathways were analyzed using the clusterProfiler package (v3.16.0; Supplementary Table 9). The pAdjustMethod was false discovery rate (FDR). The GO terms and KEGG pathways with a *q*-value < 0.1 were considered significantly enriched. We mainly focused on the pathways related to reproduction and hypothalamic function.

### RT-qPCR Validation

Some important genes (*ADCY1*, *KITLG*, *PRL*, and *FKBP5*), randomly selected mRNAs, and lncRNAs were used for validation via real-time quantitative PCR (RT-qPCR). Here, *β-actin* was used as a control. The primers were designed using Primer Premier 5 and synthesized by Qine Ke Biotech (Beijing, China; Supplementary Table 7). For RT-qPCR analysis, 1 μg of total RNA was reverse-transcribed to cDNA^TM^ following the instructions of the PrimeScript^TM^ RT Reagent Kit with gDNA Eraser (Takara, Beijing, China). The concentrations of genes and lncRNAs were diluted fourfold and used for RT-qPCR in line with the instructions of TB Green^®^ Premix Ex Taq^TM^ II (Takara, Beijing, China), performed on QuantStudio^®^ 3 (ABI, United States). All genes and lncRNAs were analyzed in triplicate. The data of each gene and lncRNA were analyzed by the 2^–ΔΔCt^ method (Livak and Schmittgen, 2001). The expression correlations between RT-qPCR and FKPM values of the RNA-seq were calculated using Pearson’s correlation coefficient by R (v.4.0.2).

## Results

### Overview of Sequencing Data in Small-Tailed Han Sheep Hypothalamus Tissue

To identify DELs during the follicular and luteal phases in the genotypes BB and WW, 12 long RNA libraries were constructed by Illumina sequencing. A total of 144.21 Gb of raw reads were obtained in this study. After filtering, the clean data of each sample reached 10.01 Gb, and the percentage of Q30 bases from each sample was higher than 90.89%, which showed that the sequencing data were highly reliable (Supplementary Table 1).

To ensure that the sequencing results did not affect the transcriptome analysis results, all of the clean reads were compared with the genome by HiSAT2 (v.2.0.5). The proportion of mapped reads and unique mapped reads of these samples reached 92.32% and 87.87%, respectively. The proportion of multiple mapped reads from the 12 groups was less than 4.01% (Supplementary Table 1). All the unique reads were divided into three libraries: extrons, introns, and intergenic.

### Identification of lncRNAs and mRNAs in Small-Tailed Han Sheep Hypothalamus Tissue

**FIGURE 1:**
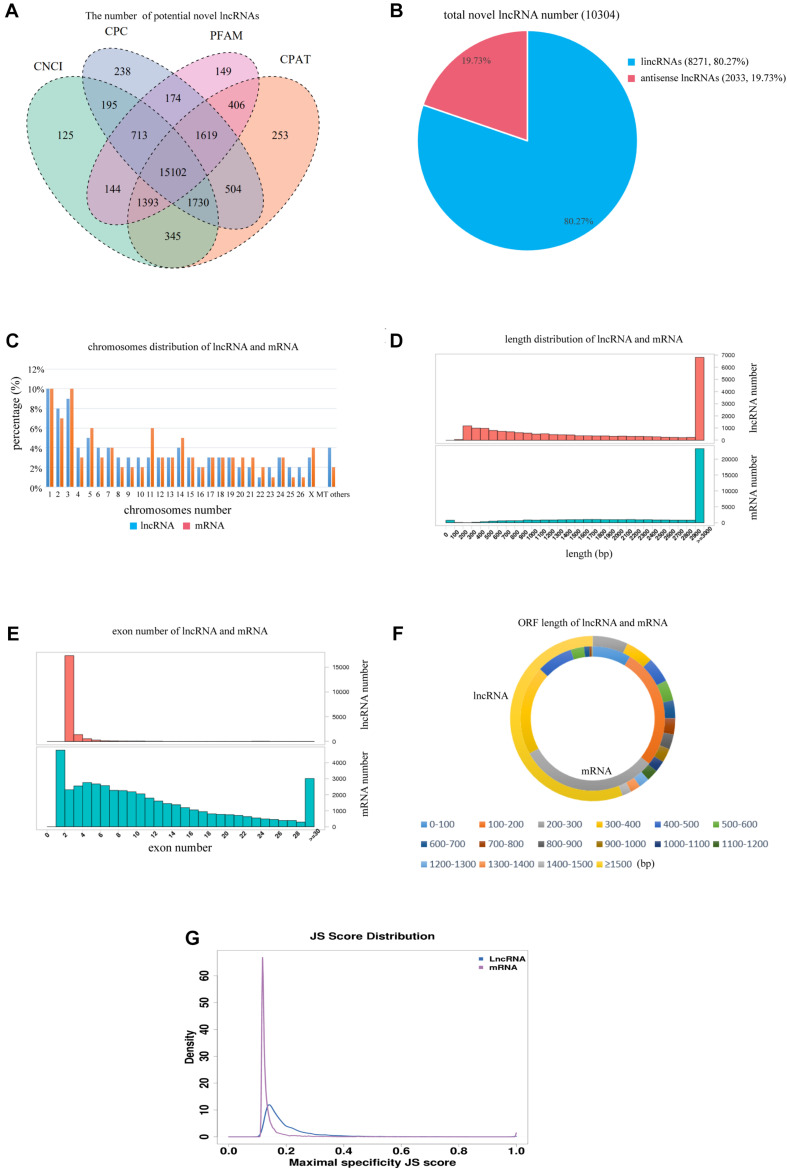
Identification of lncRNAs and mRNAs in Han sheep hypothalamus. **(A)** Venn showing the common and unique number of novel lncRNAs by four methods, including CNCI, CPC, PFAM, and CPAT. **(B)** Classification of 10,304 novel lncRNAs, including lincRNAs and antisense-lncRNAs. **(C)** Distribution of lncRNAs and mRNAs in chromosomes. **(D)** Length of lncRNAs and mRNAs. **(E)** Exon number of lncRNAs and mRNAs. **(F)** Length of ORF of lncRNAs and mRNAs. **(G)** Tissue specificity of lncRNA and mRNA.

After mapping, we combined the results of CNCI/CPC/Pfam/CPAT software to select common novel lncRNAs (Figure 1A). The results show that 15,102 novel lncRNAs were identified and that 10,304 novel lncRNAs were expressed in our samples, including 8,271 lincRNAs (80.27%) and 2,033 antisense lncRNAs (19.73%) (Figure 1B). These lncRNAs and mRNAs were randomly distributed among the X-chromosome and 26 autosomes. Overall, 500 lncRNAs (3.8%) and 522 mRNAs (2.5%) did not match any chromosomal location, and no lncRNAs are found in the mitochondria (Figure 1C). The lengths of the lncRNAs were mainly distributed in the range of about 2,900–3,000 bp, which is similar to the length distribution of mRNA transcripts in hypothalamus tissue (Figure 1D). The lncRNAs mainly contained two exons, which is significantly fewer than the exons of mRNA transcripts (Figure 1E). The average ORF length of the lncRNA transcripts (∼260 bp) was shorter than that of the mRNA transcripts (∼3,771 bp, Figure 1F). Regarding the JS score, the maximal specificity JS score of most mRNA transcripts was higher than that of most lncRNA transcripts, indicating that mRNA expression is strikingly tissue-specific compared with that of lncRNAs (Figure 1G).

### The Profiling of DELs and DEGs in Small-Tailed Han Sheep Hypothalamus

**FIGURE 2:**
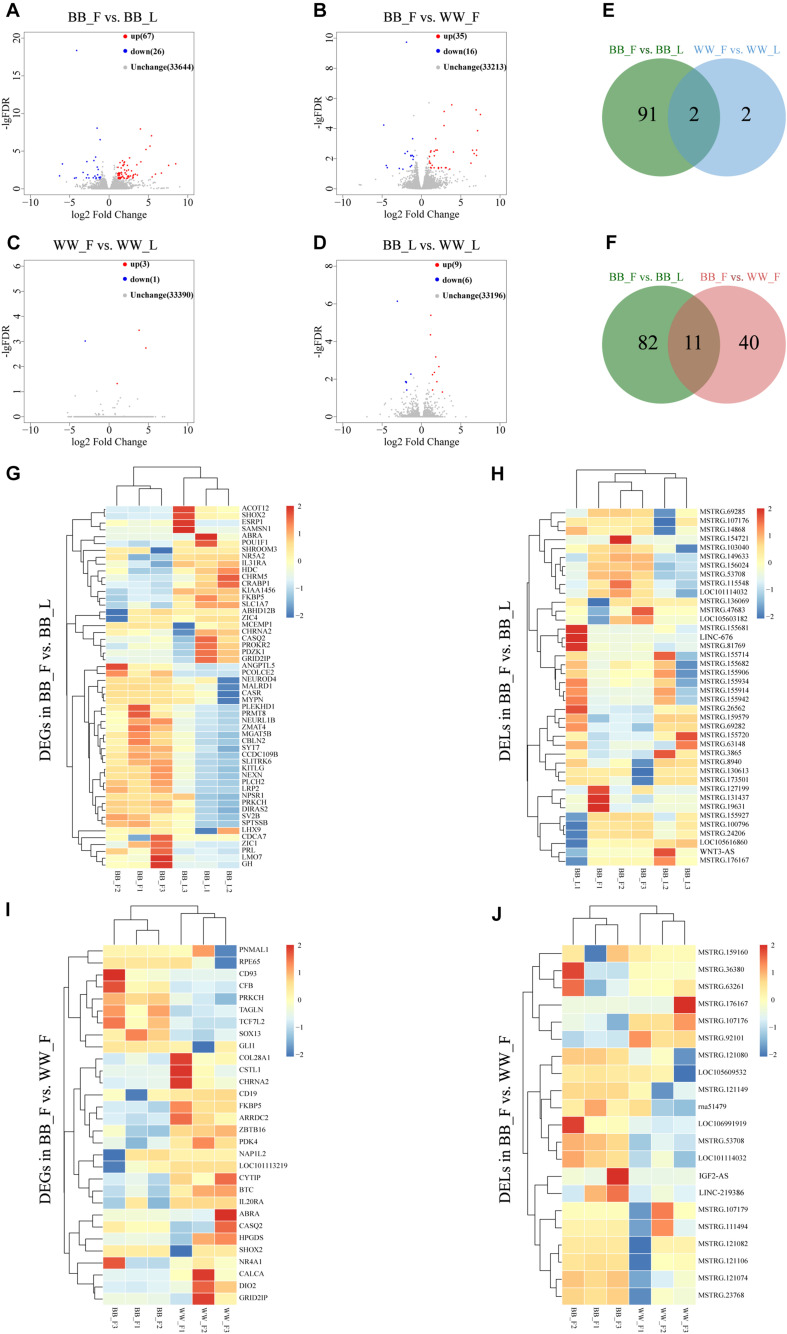
The analysis of DELs and DEGs. **(A)** Volcano showing the upregulated and downregulated genes in the BB_F vs. BB_L. **(B)** Volcano showing the upregulated and downregulated genes in the BB_F vs. WW_F. **(C)** Volcano showing the upregulated and downregulated genes WW_F vs. WW_L. **(D)** Volcano showing the upregulated and downregulated genes BB_L vs. WW_L. **(E)** Venn showing analysis of unique and shared DEGs and DELs between follicular and luteal phases. **(F)** Venn showing analysis of unique and shared DEGs and DELs between genotype BB and WW in the follicular phase. **(G)** Hierarchical cluster of DEGs in the BB_F vs. BB_L, where all the FPKM values of genes were normalized by log_2_(FPKM). **(H)** Hierarchical cluster of DELs in the BB_F vs. BB_L, where all the FPKM values of known and novel lncRNAs were normalized by log_2_(FPKM). **(I)** Hierarchical cluster of DEGs in the BB_F vs. WW_F, where all the FPKM values of genes were normalized by log_2_(FPKM). **(J)** Hierarchical cluster of DELs in the BB_F vs. WW_F, where all the FPKM values of known and novel lncRNAs were normalized by log_2_(FPKM).

Based on fold change (FC) > 2 and *q*-value < 0.05, 40 DEL transcripts and 53 DEG transcripts were identified in the BB_F vs. BB_L (genotype BB in the follicular phase vs. genotype BB in the luteal phase) comparison, 15 lncRNAs upregulated and 25 downregulated, and 11 mRNAs upregulated and 42 downregulated in the BB_F (Figures 2A,E and Supplementary Table 2). We also identified 20 DEL transcripts and 31 DEG transcripts in the BB_F vs. WW_F (genotype BB in the follicular phase vs. genotype WW in the follicular phase) comparison, four lncRNAs upregulated and 16 downregulated, and 12 mRNA upregulated and 19 downregulated in the BB_F (Figure 2B,F and Supplementary Table 2). Among them, four DEL transcripts and seven DEG transcripts overlapped, which are considered the key lncRNAs and genes affected by the involvement of *FecB* mutation in follicular development (Figure 2F). Overall, one DEL and three DEG transcripts were identified in the WW_F vs. WW_L (genotype WW in the follicular phase vs. genotype WW in the luteal phase) comparison, one lncRNA upregulated and three mRNA downregulated in the WW_F (Figures 2C,E and Supplementary Table 2). In addition, six DEL transcripts and nine DEG transcripts were identified in the BB_L vs. WW_L (genotype BB in the luteal phase vs. genotype WW in the luteal phase) comparison, four lncRNAs upregulated and two downregulated, and two mRNAs upregulated and seven downregulated in the BB_L (Figure 2D and Supplementary Table 2). Owing to there being fewer DEGs and lncRNAs screened out in the WW_F vs. WW_L and BB_L vs. WW_L comparisons, the following discussion does not focus on them. To search for similarities and differences, systematic clustering analysis was used to compare the expression patterns of DEGs and DELs of different phases and genotypes (Figures 2G–J).

### Functional Analysis of DEGs

All DEGs are tested in GO terms to clarify their biological implications. The top enriched terms of DEGs from the BB_F vs. BB_L comparison were junctional membrane complex, growth factor complex, and luteinizing hormone secretion. In addition, some of the identified terms were related to follicular–luteal transition, including gonadotropin secretion, circadian rhythm, and hormone metabolic process (Supplementary Table 4). The top enriched terms of DEGs from the BB_F vs. WW_F comparison were cis–*trans* isomerase activity, neurotransmitter receptor activity, and dopamine receptor binding. Notably, some of the terms were related to the regulation of hormones, such as steroid hormone receptor activity, hormone transport, peptide hormone secretion, and hormone secretion (Supplementary Table 4).

**FIGURE 3:**
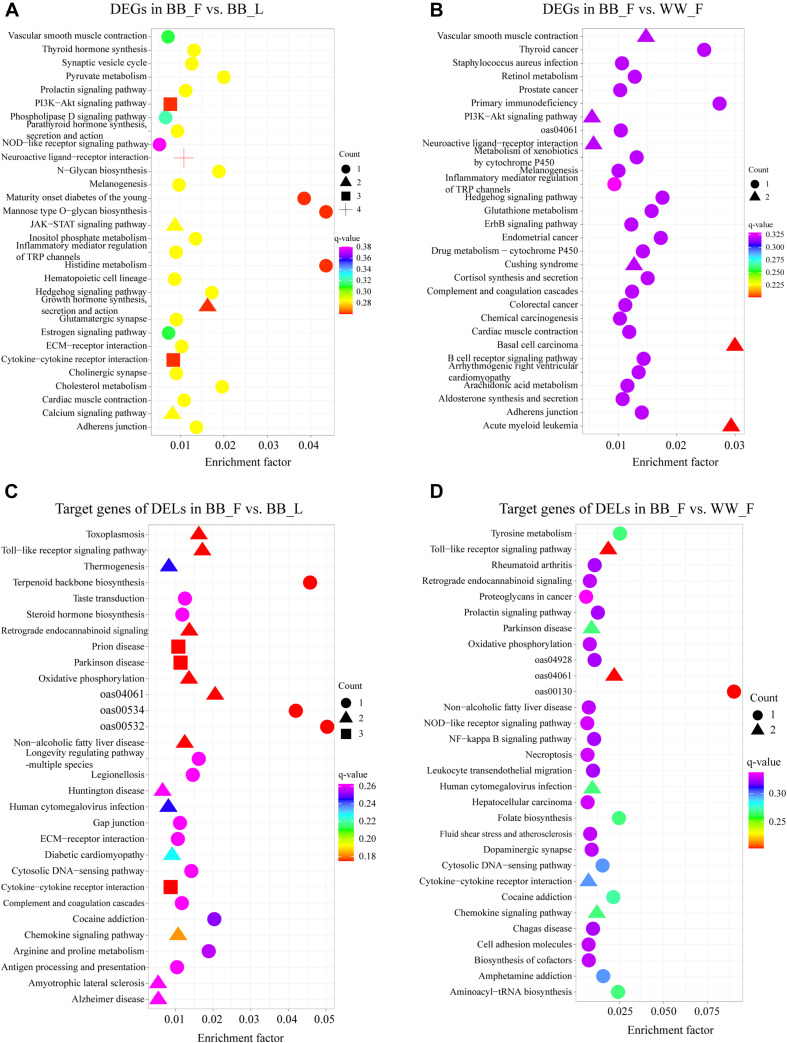
Top 30 enriched KEGG pathways related to hypothalamic function and reproductive process of DEGs and target genes of DELs. Enrichment factor is the ratio of differentially expressed gene numbers annotated in this pathway terms to all gene numbers annotated in this pathway term. **(A)** KEGG enrichment pathways for DEGs in BB_F vs. BB_L. **(B)** KEGG enrichment pathways for DEGs in BB_F vs. WW_F. **(C)** KEGG enrichment pathways for potential target genes in BB_F vs. BB_L. **(D)** KEGG enrichment pathways for potential target genes in BB_F vs. WW_F.

Based on the KEGG enrichment analysis, the DEGs of the BB_F vs. BB_L comparison were annotated to 36 pathways (Supplementary Table 4). The top 30 KEGG pathways are shown in Figure 3A. Notably, some pathways were associated with follicular–luteal transition, such as the neuroactive ligand–receptor interaction, melanogenesis, estrogen, MAPK, PI3K–Akt, and prolactin signaling pathways. In addition, the DEGs of the BB_F vs. WW_F comparison were annotated to 48 pathways. The top 30 pathways were related to hypothalamic function and fecundity, including the neuroactive ligand–receptor interaction, estrogen, MAPK, Wnt, ErbB, and Hippo signaling pathways as well as several hormone-related pathways like estrogen, melanogenesis, and GnRH secretion (Figure 3B and Supplementary Table 4).

### Screening of Potential Functional lncRNAs Involved in Small-Tailed Han Sheep Reproduction

**FIGURE 4:**
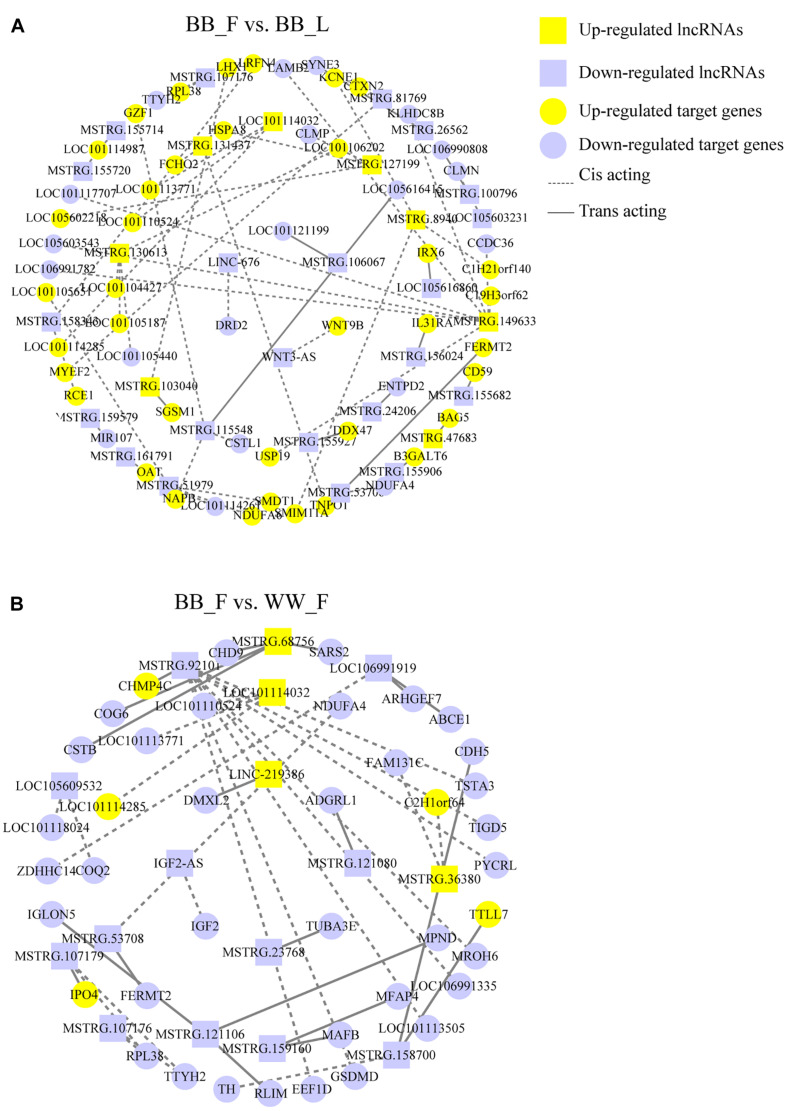
DELs interaction with protein-coding genes consist of network. **(A)** In the BB_F vs. BB_L, 29 DELs are *cis-*acting or *trans-*acting with 58 mRNAs, which build the interactive network. The yellow and purple colors are for upregulated and downregulated lncRNA and target genes, respectively. Rectangles and ellipses are for lncRNA and target gene, respectively. The dotted and straight lines are for *cis-*acting and *trans-*acting, respectively. **(B)** In the BB_F vs. WW_F, 14 DELs are *cis-*acting or *trans-*acting with 42 mRNAs, which build the interactive network.

To further explore the lncRNAs related to the hypothalamic function and fecundity of small-tailed Han sheep, an interaction network of the lncRNAs and their potential *trans-* and *cis-*target genes was constructed. In the BB_F vs. BB_L comparison, three known lncRNAs corresponded to five target genes, and 26 novel lncRNAs corresponded to 53 target genes. These DELs and their target genes were particularly associated with hypothalamic function and reproduction. The target genes (*DRD2*, *WNT9B*, *NAPB*, and *CLMN*) of these DELs (LINC-676, WNT3-AS, MSTRG.11548, and LOC105603231) were particularly associated with GO terms, including regulation of neurotransmitter transport and neuron development, as well as KEGG pathways, including neuroactive ligand–receptor interaction, steroid hormone biosynthesis, dopaminergic synapse, estrogen, cAMP, and MAPK signaling pathways (Figure 3C and Supplementary Table 5). DRD2, WNT9B, NAPB, and CLMN are potential *cis-*elements of LINC-676, WNT3-AS, MSTRG.11548, and LOC105603231, respectively, acting through sequence complementarity (Figure 4A and Supplementary Table 6). In the BB_F vs. WW_F comparison, three known lncRNAs corresponded to eight target genes, and 11 novel lncRNAs corresponded to 34 target genes. The potential target gene *DMXL2* was *trans-*regulated by LINC-219386 and *IGF2* was *cis-*regulated by IGF2*-*AS, which were particularly associated with GO terms, including reproductive process and hormone activity and KEGG pathways including prolactin, PI3K–Akt, and MAPK signaling pathways (Figures 3D, 4B and Supplementary Tables 5, 6).

### Validation of RNA Sequencing Using RT-qPCR

**FIGURE 5:**
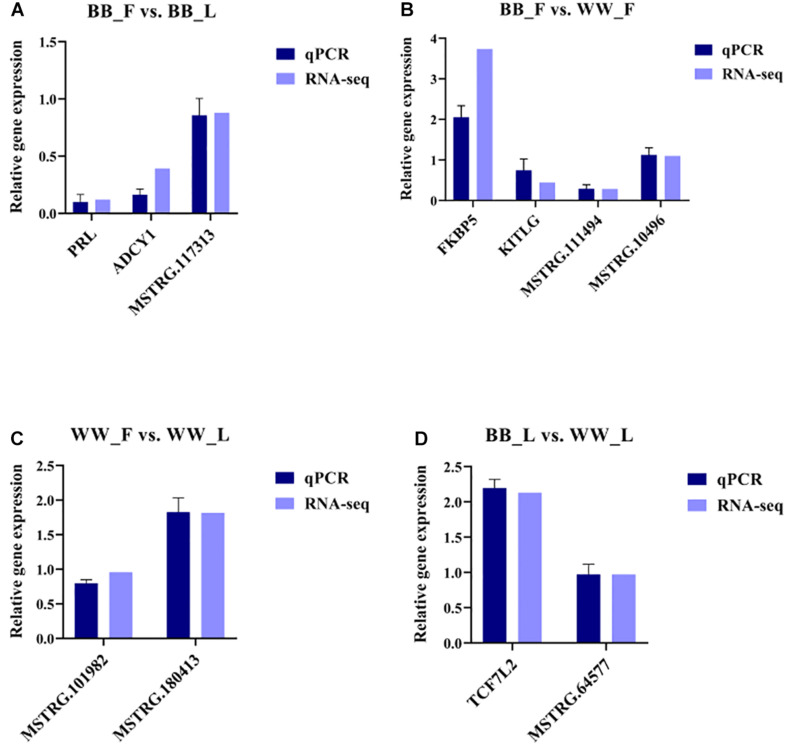
Validation of RNA-seq data by RT-qPCR. RT-qPCR and RNA-seq data are presented as relative gene expression. **(A)** Selected genes and lncRNAs from BB_F vs. BB_L are validated by RT-qPCR and RNA-seq, respectively. **(B)** Selected genes and lncRNAs from BB_F vs. WW_F are validated by RT-qPCR and RNA-seq, respectively. **(C)** Selected genes and lncRNAs from WW_F vs. WW_L are validated by RT-qPCR and RNA-seq, respectively. **(D)** Selected genes and lncRNAs from BB_F vs. WW_L are validated by RT-qPCR and RNA-seq, respectively.

To validate the RNA-seq data, some important genes (*ADCY1*, *KITLG*, *PRL*, and *FKBP5*) and randomly selected mRNAs and lncRNAs were examined by RT-qPCR, and the relative gene expression was calculated using the 2^–Δ^ ^Δ^ ^*Ct*^ method (Figure 5). The obtained results indicate that the data regarding the mRNA and lncRNA expression levels are reliable. Meanwhile, the results of Pearson’s correlation analysis of all genes show that there was a strong positive correlation between the RT-qPCR and RNA-seq data (corr > 0.93, *p* < 0.05).

## Discussion

In the domestic sheep, the hypothalamic hormones and factors control different glandular hormone secretions that are involved in the whole reproductive process. During follicle development, *FecB* mutation regulates the response of GCs and oocytes to FSH and LH by impairing the BMP signaling pathway, which leads to the accelerated maturation and ovulation of preantral follicles (Fabre et al., 2006). That is, the *FecB* gene is closely related to sheep fertility. More and more evidence also indicates that lncRNAs function in the sheep reproductive process (Miao et al., 2016, 2017; Feng et al., 2018; Li et al., 2020). Nevertheless, the current research on lncRNA is principally centered on the ovary, ignoring the role of FecB response changes from folliculogenesis to ovulation, such as changes in hypothalamic GnRH secretion and its regulation of downstream hormones (FSH, LH, and PRL). The present study focuses on hypothalamus tissue at the transcriptome level, to investigate the function of the key genes and lncRNAs on that *FecB* mutation increases ovulation rate via the hypothalamic–pituitary–ovarian axis.

Gonadotropin-releasing hormone released by the hypothalamus is responsible for neuroendocrine modulation in assorted physiological states (Ciechanowska et al., 2010). GnRH has two molecular forms: GnRH1 and GnRH2. GnRH1 drives mammalian reproduction by regulating the gonadotropins, and the *GnRH2* gene was shown to be inactivated in the sheep in a previous study (Desaulniers et al., 2017). In our study, the molecular forms of GnRH were characterized, but their expression levels were very low. There were no differences in GnRH levels between the *FecB* genotypes and estrus states. Hypothalamus transcriptome analysis identified 52 DEGs between the follicular and luteal phases, almost all of which were only significantly differentially expressed in the genotype BB ewes compared with the wild type. These DEGs were involved in neural cellular differentiation and proliferation, synapse formation, and the receipt of neuronal information, such as neuralized E3 ubiquitin protein ligase 1B (*NEURL1B*), synaptic vesicle glycoprotein 2B (*SV2B*), and cerebellin 2 precursor (*CBLN2*) (Hirai et al., 2005; Morgans et al., 2009; Pape et al., 2010), which were revealed to be highly expressed in the luteal phase, and neuropeptide S receptor 1 (*NPSR1*) was highly expressed in the follicular phase. Given that these genes have hypothalamus-specific expression, we hypothesized that they might modulate neuronal messages traveling from the hypothalamus to the pituitary in the follicular–luteal transition, mediated by the *FecB* gene. In addition, the DEG *PRL* was identified, which was enriched in the most pathways related to hypothalamic function and follicular development, including the neuroactive ligand–receptor interaction, PI3K–Akt, and prolactin signaling pathways. PRL is demonstrated to be expressed in the hypothalamus (Roselli et al., 2008; Zou et al., 2019). That is, hypothalamic PRL mainly functions in the transmission of neural signals and the synthesis and secretion of reproductive hormones.

Tian et al. (2020) reveals 139 DEGs from the hypothalamus among different FecB genotypes, which were mainly enriched in the neuroactive ligand–receptor interaction and cAMP signaling pathways. This work reveals similar DEG-related enriched pathways to that study. Subsequently, seven DEGs were found to overlap between the BB_F vs. BB_L and BB_F vs. WW_F comparisons, which were involved in the follicular–luteal transition regulated by *FecB* mutation. The DEG FKBP Prolyl Isomerase 5 (*FKBP5*) was directly regulated by progestin that activates the *FKBP5* promoter (Kester et al., 1997). Overexpression of hypothalamic *FKBP5* might attenuate progestin responsiveness in hormone-conditioned cells (Hubler et al., 2003). That is, the increased progesterone level may induce *FKBP5* mRNA in the luteal phase. However, our results show that the expression level of *FKBP5* was significantly higher in BB_F than in WW_F, which might be explained by the impact of *FecB* mutation. Moreover, it also indicates that the inhibitory effect of progesterone on pituitary secretion is curbed in the follicular phase; that is, *FKBP5* might tell the pituitary to pump out more FSH. The KIT Ligand (*KITLG*) plays a pivotal role in the development, migration, and differentiation of many different cell types in the body, including melanocytes, germ cells, and blood cells (Morrison-Graham and Takahashi, 1993). In addition, Melanophilin (*MLPH*) is a Rab27a- and myosin-binding protein that regulates the melanosome transport (Hume et al., 2007). In terms of KEGG analysis, *KITLG* was found to be particularly associated with hormone-related pathways, such as melanogenesis, oxytocin, and GnRH secretion, which are involved in female reproduction. That is, KITLG is involved in the secretion of GnRH by regulating melatonin in the follicular phase because of the modification of steroid hormone feedback at the HPO axis (Malpaux et al., 1998; Díaz et al., 1999). Nonetheless, the level of *KITLG* was found to be significantly lower in BB_F than in WW_F; however, the results for this gene may be unreliable because its expression level was not uniformly distributed across the three biological repeats in genotype WW.

In this study, 10,304 novel lncRNA transcripts were screened out. The number of lncRNAs was similar to those in previous studies on sheep (13,148 and 9082, respectively; Ma et al., 2018; Zhao et al., 2020) but higher than that in mouse (1112) (Ma et al., 2019). To better understand the function of lncRNAs in the follicular–luteal transition, a lncRNA–mRNA network was established. According to the KEGG pathway analysis, some potential target genes are involved in dopaminergic synapse and neuroactive ligand–receptor interaction and the cAMP signaling pathway, and these warrant further discussion, including dopamine receptor D2 (*DRD2*) and Wnt family member 9B (*WNT9B*). *DRD2*, a *cis-*element of the lncRNA LINC-676, downregulates the synthesis of GnRHRs to curb GnRH activity and contains basal GnRH-induced gonadotropins released from the pituitary (Yaron et al., 2003; Levavi-Sivan et al., 2004). *WNT9B*, a *cis-*element of the lncRNA WNT3-AS, might function in follicular development by recruiting β-catenin like *WNT2* (Wang et al., 2009). Furthermore, our sequencing data reveals that the expression of these genes and lncRNAs was decreased while that of *WNT9B* and MSTRG.156432 was increased in the follicular phase compared with their levels in the luteal phase. That is, in follicular–luteal transition, the lncRNAs showed trends of shifting from low to high expression. Meanwhile, their potential target genes were mainly related to hormone synthesis and secretion, implying that the expression of lncRNAs may be related to the fluctuation of hormone secretion. Our previous study on different phases of wild-type sheep identified that DELs were involved in the amino acid metabolic process, and the target gene *WNT7A* (Wnt Family Member 7A) may participate in the sheep reproductive process (Zhang et al., 2019). Interestingly, the results from sheep with FecB mutation in different phases indicate that DELs are mainly associated with the hormone metabolic process and also reveal that one of the members of the WNT gene family, *WNT9B*, is likely to be involved in the follicular–luteal transition. That is, there were differences of regulatory elements and WNT gene family members involved in the reproductive process of the follicular–luteal transition between wild-type (*WNT7A*) and *FecB* mutant sheep (*WNT9B*).

Miao et al. (2016) identify several ovarian lncRNAs that may be related to the follicular development by regulating target genes related to TGF-β and OXT signaling pathways that were differentially expressed between *FecB* mutant homozygous and wild-type small-tailed Han sheep. However, we reveal that hypothalamic lncRNAs (LINC-219386 and IGF2-AS) may be associated with reproductive impairment by controlling their target genes (*DMXL2* and *IGF2*) related to the GnRH signaling pathway in follicular development against a background of *FecB* mutation involved in follicular development and ovulation. DMXL2 and IGF2 play similar roles in response to GnRH by stimulating GnRHR and FSH synthesis (Tata et al., 2014; Ye et al., 2017). Wahab et al. (2017) report that low expression levels of *DMXL2* exhibit a complicated neurological phenotype along with an absence of the gonadotropic axis and abnormal glucose metabolism due to the significant loss of GnRH neurons. That is, DMXL2 and IGF2 act as factors that co-orchestrate the neuroendocrine activation of reproduction and puberty. In our study, LINC-219386, *DMXL2*, IGF2-AS, and *IGF2* were all upregulated in the genotype BB compared with their levels in WW in the follicular phase, indicating that *FecB* mutation is likely to increase LINC-219386 and IGF2-AS expression, modulating their target genes *DMXL2* and *IGF2* to produce more GnRH during follicular development. This explains why *FecB* mutated ewes produced more mature follicles.

In our study, some novel lncRNAs acting on target genes were revealed to affect hypothalamic function and the reproductive process via hormones and other regulatory factors, such as LINC-676, WNT3-AS, and IGF2-AS *cis-*acting on *DRD2*, *WNT9B*, and *IGF2*, LINC-219386 *trans-*acting on *DMXL2*, respectively. Nevertheless, one limitation of this study is that we barely provided indirect experimental results to deduce the functional connections among components of the lncRNA–mRNA network, preventing definitive proof of the obtained findings. In future research, we will give evidence of our predictions, the link between lncRNAs, and their potential target genes and elucidate how these lncRNAs function in sheep fertility.

## Conclusion

This hypothalamus transcriptome analysis reveals the expression profiles of the hypothalamus with the *FecB* mutation at different estrus states. Upon comparing the two estrus states, the identified DEGs and the potential target genes of the DELs were found to be associated with follicular development. Upon combining the DEG and DEL data sets screened from different estrus states and genotypes, the DEGs and the potential target genes were shown to act on the hypothalamus, such as by increasing GnRH neurons to regulate the ovulation in a manner mediated by *FecB* mutation. Our findings provide new insights into the endocrine mechanisms involved in follicle development of *FecB* mutated sheep and offer candidate lncRNAs and genes linked to hypothalamic function and the reproductive process.

## Data Availability Statement

The datasets presented in this study can be found in online repositories. The names of the repository and accession number can be found below: National Center for Biotechnology Information (NCBI) Sequence Read Archive (SRA) https://www.ncbi.nlm.nih.gov/sra/, PRJNA672275.

## Ethics Statement

The animal study was reviewed and approved by the Animal Ethics Committee of the IAS-CAAS (No. IAS 2019-49). Written informed consent was obtained from the owners for the participation of their animals in this study.

## Author Contributions

XG, XW, and MC conceived and designed the experiments. XG and XW performed the experiments. SC analyzed the data and wrote the manuscript with input from the other authors. All authors contributed to the article and approved the submitted version.

## Conflict of Interest

The authors declare that the research was conducted in the absence of any commercial or financial relationships that could be construed as a potential conflict of interest.

## Footnotes

1 http://ccb.jhu.edu/software/stringtie/gff.shtml

2 http://cole-trapnell-lab.github.io/cufflinks/cuffcompare/index.html#transfrag-class-codes

## Supplementary Material

Supplementary Table 1 Alignment of statistical results of reads.

Supplementary Table 2 The results of differentially expressed mRNAs.

Supplementary Table 3 The results of differentially expressed lncRNAs.

Supplementary Table 4 DEG GO and KEGG enrichment analysis.

Supplementary Table 5 Target genes of DEL GO and KEGG enrichment analysis.

Supplementary Table 6 The lncRNA-mRNA network.

Supplementary Table 7 Primer sequence and product size of mRNAs and lncRNAs for RT-qPCR.

Supplementary Table 8 The sequence of DE lncRNAs.

Supplementary Table 9 The code of DESeq2 and clusterProfiler.
